# Draft Genome Sequence of *Lactiplantibacillus plantarum* IMI 507026

**DOI:** 10.1128/mra.00305-22

**Published:** 2022-05-16

**Authors:** Ivana Nikodinoska, Jenny Makkonen, Daniel Blande, Colm Moran

**Affiliations:** a Alltech European Headquarters, Sarney, Dunboyne, Meath, Ireland; b Biosafe – Biological Safety Solutions Ltd., Kuopio, Finland; c Regulatory Affairs Department, Alltech SARL, Vire, France; University of Arizona

## Abstract

Here, we announce the draft genome sequence of Lactiplantibacillus plantarum isolated from corn silage in Nicholasville, KY. L. plantarum IMI 507026 is deposited in the Centre for Agriculture and Bioscience International (CABI) Culture Collection with the accession number IMI 507026.

## ANNOUNCEMENT

Members of lactic acid bacteria are commonly used to support more sustainable and efficient food production (1). Lactiplantibacillus plantarum is an important silage inoculant due to the quick lactic acid production and silage pH reduction, together with low dry matter losses (2, 3). Here, we describe the draft genome of *Lactiplantibacillus plantarum* IMI 507026.

The corn silage sample was obtained from a farm located in Kentucky and after homogenization with 0.85% NaCl solution (1:10) was plated onto deMan-Rogosa-Sharpe (MRS) agar and incubated at 37°C for 48 h under anaerobic conditions. The isolated colonies were grown anaerobically in 10 mL MRS broth and incubated at 37°C for 16 to 17 h; after, genomic DNA extraction was performed according to the sample preparation and lysis protocol described for Gram-negative and some Gram-positive bacterial samples in the genomic DNA handbook (Qiagen), and DNA was purified according to the Genomic-Tip 100/G procedure (Qiagen). DNA was submitted to Eurofins Genomics (Constance, Germany) for library preparation (NEBNext Ultra II Fragmentation System DNA library prep kit; New England BioLabs, San Diego, CA) and whole-genome sequencing (NovaSeq 6000, Illumina), producing 2 × 150-bp paired-end reads. The sequencing produced 6,325,888 raw reads. The reads were trimmed using Trimmomatic v0.38.1 (4) and assembled using Unicycler v0.4.8 (5), generating 35 contigs with a genome length of 3,233,448 bp, 538-fold coverage, GC content of 44.51%, and an *N*_50_ value of 418,725 bp. The annotation with NCBI Prokaryotic Genome Annotation Pipeline (PGAP) v6.0 (6) predicted 3,053 total genes number, 2,939 coding genes, 59 tRNA genes, and 2 rRNA genes. The IMI 507026 genome showed a Mash (7) (software MinHash v0.1.1) distance of 0.000236 and a calculated OrthoANI (8) (software OrthoANI v1.40) value of 99.994% with *L. plantarum* JDM1 (accession number CP001617.1).

Searches for antimicrobial resistance genes, toxin and virulence factor genes, and plasmid presence in the genome were performed according to a previously described procedure, without modifications (9). No acquired antimicrobial resistance genes of concern that were above 80% identity and 70% coverage were found. In addition, no genes encoding potential virulence or pathogenicity factors were detected. The bioinformatic analysis identified two contigs as potential plasmids. PlasmidFinder matches were found with contig 21 (3,884 bp) to *L. plantarum* subsp. *plantarum* P-8 plasmid LBPp6 (8,686 bp) and contig 23 (2,111 bp) to *L. plantarum* strain PA18 plasmid pR18 (3,211 bp).

### Data availability.

All data are available under BioProject accession number PRJNA814906. The raw reads were deposited at the SRA under accession number SRR18309003. The GenBank accession number is JALBGE000000000.
